# Identification and characterization of regulatory pathways involved in early flowering in the new leaves of alfalfa (*Medicago sativa* L.) by transcriptome analysis

**DOI:** 10.1186/s12870-020-02775-9

**Published:** 2021-01-06

**Authors:** Dongmei Ma, Bei Liu, Lingqiao Ge, Yinyin Weng, Xiaohui Cao, Fang Liu, Peisheng Mao, Xiqing Ma

**Affiliations:** 1 Breeding Base for State Key Laboratory of Land Degradation and Ecological Restoration in Northwest China/ Ministry of Education Key Laboratory for Restoration and Reconstruction of Degraded Ecosystems in Northwest China, Ningxia University, Yinchuan, 750021 China; 2 College of Grassland Science and Technology, China Agricultural University, Beijing, 100193 China; 3 National Animal Husbandry Service, Maizidian Street, North Nongzhan Road, Chaoyang District, Beijing, 100125 China

**Keywords:** Alfalfa, Flowering time, New leaves, Hormone, Transcriptome

## Abstract

**Background:**

Alfalfa (*Medicago sativa* L.) is a perennial legume extensively planted throughout the world as a high nutritive value livestock forage. Flowering time is an important agronomic trait that contributes to the production of alfalfa hay and seeds. However, the underlying molecular mechanisms of flowering time regulation in alfalfa are not well understood.

**Results:**

In this study, an early-flowering alfalfa genotype 80 and a late-flowering alfalfa genotype 195 were characterized for the flowering phenotype. Our analysis revealed that the lower jasmonate (JA) content in new leaves and the downregulation of JA biosynthetic genes (i.e. lipoxygenase, the 12-oxophytodienoate reductase-like protein, and salicylic acid carboxyl methyltransferase) may play essential roles in the early-flowering phenotype of genotype 80. Further research indicated that genes encode pathogenesis-related proteins [e.g. leucine rich repeat (LRR) family proteins, receptor-like proteins, and toll-interleukin-like receptor (TIR)-nucleotide-binding site (NBS)-LRR class proteins] and members of the signaling receptor kinase family [LRR proteins, kinases domain of unknown function 26 (DUF26) and wheat leucine-rich repeat receptor-like kinase10 (LRK10)-like kinases] are related to early flowering in alfalfa. Additionally, those involved in secondary metabolism (2-oxoglutarate/Fe (II)-dependent dioxygenases and UDP-glycosyltransferase) and the proteasome degradation pathway [really interesting new gene (RING)/U-box superfamily proteins and F-box family proteins] are also related to early flowering in alfalfa.

**Conclusions:**

Integrated phenotypical, physiological, and transcriptomic analyses demonstrate that hormone biosynthesis and signaling pathways, pathogenesis-related genes, signaling receptor kinase family genes, secondary metabolism genes, and proteasome degradation pathway genes are responsible for the early flowering phenotype in alfalfa. This will provide new insights into future studies of flowering time in alfalfa and inform genetic improvement strategies for optimizing this important trait.

**Supplementary Information:**

The online version contains supplementary material available at 10.1186/s12870-020-02775-9.

## Background

Alfalfa (*Medicago sativa* L.) is a perennial leguminous forage extensively planted as livestock feed around the world due to the high crude protein and nutrition contents in leaves [1, 2]. While flowering time is an important determinant of harvesting time, which contributes to forage quality, silage, yield, and production in alfalfa [3]. Early flowering can improve the number of cuttings, forage yield, and year-round production of alfalfa; it can also help to avoid the rainy season or drought stress for seed production, and enhance winter hardiness related to the temperate semi-arid continental climate in China [4]. Despite the importance, the underlying molecular mechanisms involved in flowering time control in alfalfa remain elusive.

Past endeavors of flowering time studies discovered six major regulatory pathways in model plants Arabidopsis, rice (*Oryza.sativa* L.), wheat (*Triticum aestivum* L.), and barley (*Hordeum vulgare* L.) [5, 6]. These pathways can be categorized into two groups: the photoperiod, vernalization, and ambient pathways that respond to environmental conditions, and the autonomous, gibberellin, and age pathways that respond to endogenous cues associated with the up/downregulation of floral integrator genes, including *FLOWERING LOCUS T* (*FT*), *FLOWERING LOCUS C* (*FLC*), *CONSTANS* (*CO*) and *SUPPRESSOR OF OVEREXPRESSION OF CONSTANS1* (*SOC1*) [7–9]. Furthermore, small non-coding RNAs and epigenetic pathways play important roles in regulating flowering time in plants [10]. For instance, in the plant adult phase, miR156 targets *SQUAMOSA PROMOTER BINDING-LIKE* (*SPL*) and induces flowering by upregulation expression of flower-promoting genes in the shoot apex [11], and FLOWERING LOCUS D, histone demethylase, downregulates *FLC* expression associated with mediating H3K4 demethylation and therefore promotes flowering in Arabidopsis [12]. An earlier study also indicated that jasmonate-activated (JA) transcription factors *MYC2*, *MYC3*, and *MYC4* negatively regulate flowering time by interacting with *FT*, and exogenous JA could delay flowering time in Arabidopsis [13]. Applying exogenous abscisic acid (ABA) or inducing the overexpression of *ABI5*, an *ABSCISIC ACID-INSENSITIVE MUTANT 5*, through *FLC* expression can also trigger delayed flowering in Arabidopsis [14, 15]. In addition, receptor-like kinase (RLK) family proteins play a key role in the perception of internal and external signals involved in flowering time regulation [16]. For instance, FLOR1, a leucine-rich repeat (LRR) protein expressed in the shoot meristem, physically interacts with AGAMOUS (AG, a MADS-box transcription factor) and is induced in the early inflorescence meristem, and the Arabidopsis *flor1* mutant exhibits a delayed flowering phenotype [17]. CURVY1, a novel receptor-like protein kinase, also responds to flowering time and seed production in Arabidopsis [18]. Furthermore, the ubiquitin-proteasome system is a major catabolic pathway involved in plant growth, development, and physiological processes, including floral development. Tagging of the target proteins with ubiquitin is mainly performed by E1 (ubiquitin-activating enzyme), E2 (ubiquitin-conjugating enzyme), and E3 (ubiquitin-protein ligase enzyme), followed by degradation mediated by the 26S proteasome [19]. For example, PUB13, a U-box protein possessing E3 ubiquitin ligase, physically interacts with HFR1 (long hypocotyl in far-red light1) to regulate photomorphogenesis and flowering time in Arabidopsis [20]. However, whether their alfalfa homologs participate in flowering time regulation is not clear.

Previous studies using the shoot tip or leaf samples of alfalfa plants of different flowering stages have indicated that proteins involved in carbohydrate metabolism, phenylpropanoid biosynthesis and immunity are responsive to flowering time [4, 21]. However, little is known about the underlying molecular mechanisms associated with flowering time control in new leaves, which represent the transition from the vegetative to the floral phase in alfalfa. It has been shown that leaf development and floral organ initiation are controlled by common homeotic factors [22]. For instance, knocking down the *AG* gene resulted in the reprogramming of leaf development and affected the expression of key regulatory genes (*TRIPTYCHON*, *APETALA3* and *PISTILLATA*) in Arabidopsis leaves [22, 23]. Therefore, we hypothesize that these metabolic genes may play essential roles in new leaves of alfalfa during the early flowering stage. Here, integrated phenotypical, physiological, and transcriptome analyses showed that hormone biosynthesis and signaling pathway genes, pathogenesis-related (PR) genes, signaling receptor kinase family genes, secondary metabolism genes, and proteasome degradation pathway genes might contribute to the early flowering phenotype in alfalfa.

## Results

### Phenotypes and endogenous hormone contents

**Fig. 1:**
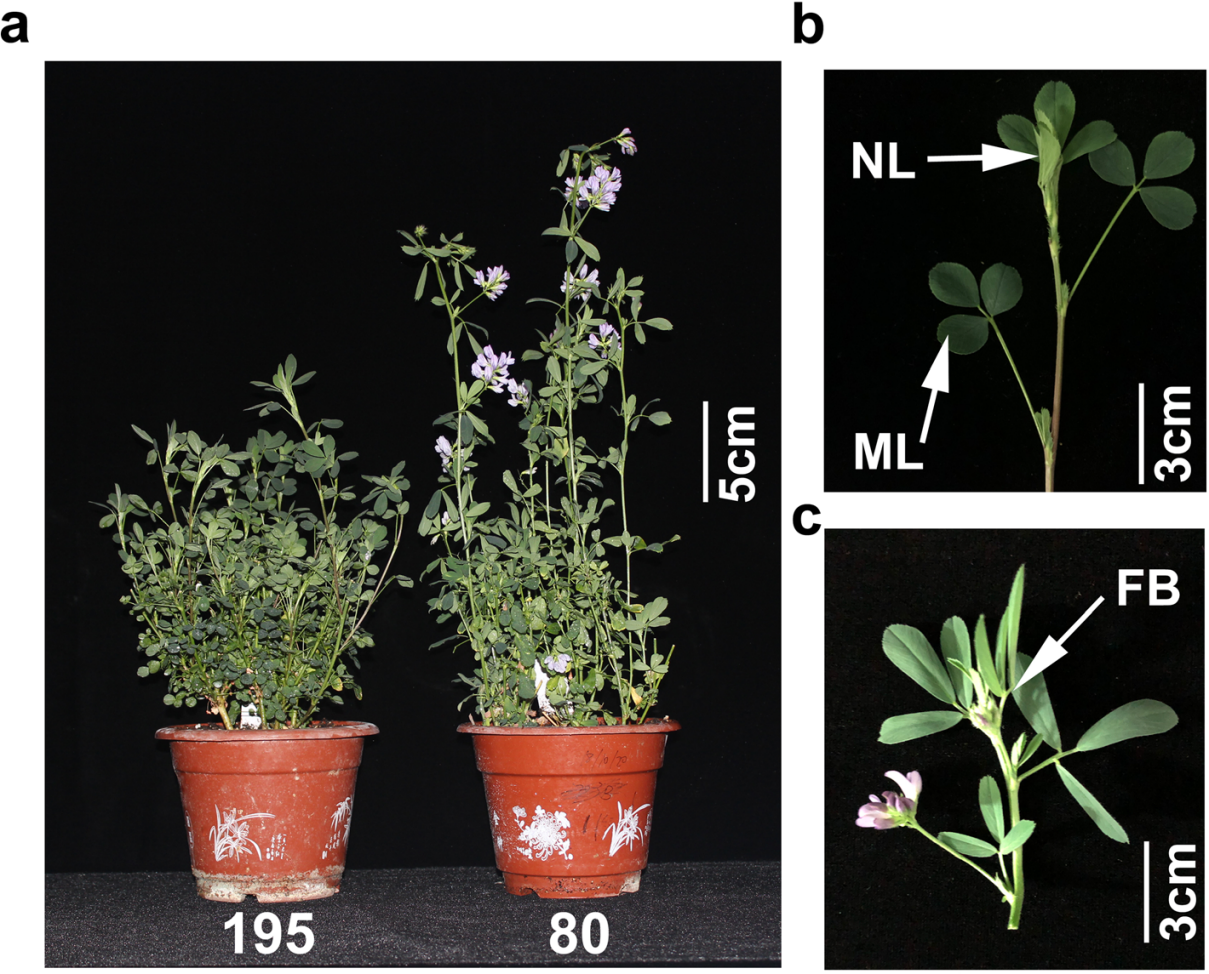
Phenotypic characterization of genotypes 195 and 80 of alfalfa. a, The phenotype of genotype 195 and 80 plants grown in a controlled climate chamber at the time of sampling (25 days after plants). b and c, Tissues harvested for transcriptome analysis. ML, mature leaves; NL, new leaves including the apical meristem; FB, flower buds

**Fig. 2:**
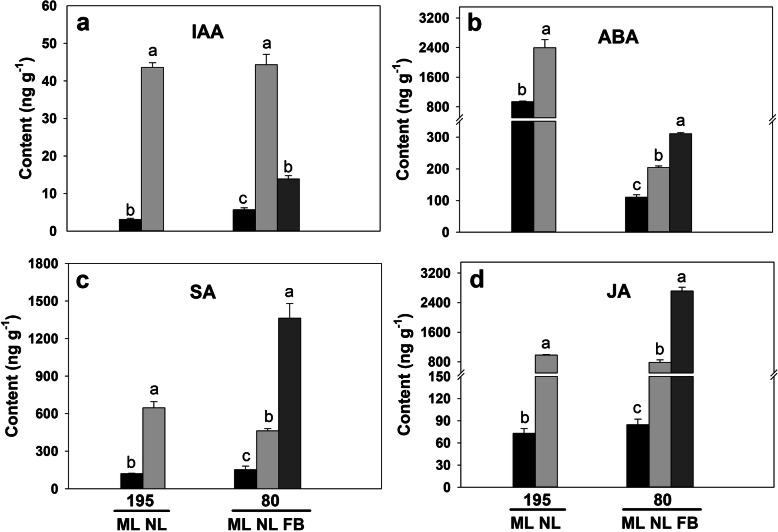
Contents of endogenous IAA (**a**), ABA (**b**), SA (**c**), and JA (**d**) in genotype 195 and 80 at the time of sampling. Different letters indicate significant differences among ML (mature leaves), NL (new leaves including the apical meristem), and FB (flower buds) based on the LSD test (*P* < 0.05). Data are shown as the mean ± standard deviation (SD) of three replicates

For the flowering time assays under controlled growth chamber conditions, plants were trimmed to maintain the same height. Genotype 80 first flowered at 25 days after planting with a mean plant height of 30.95 cm, when the late-flowering genotype 195 had an average plant height of only 19.73 cm (Fig. 1a). Genotype 195 then flowered at 50 days after planting. Endogenous hormone contents in mature leaves (ML), new leaves (NL) including apical meristem, and flower buds (FB) were determined in both genotypes at 25 days after planting when genotype 80 flowered (Fig. 1b and c; Fig. 2a-d). We observed significantly higher auxin (IAA), ABA, salicylic acid (SA), and JA contents in NL compared with ML in both genotypes, while, the differences in hormone levels between NL and ML was smaller in genotype 80 than in 195 (Fig. 2a-d). In addition, except for IAA, hormone contents in FB were significantly higher than those in ML or NL in genotype 80 (Fig. 2a-d). Together, these results suggest a higher translocation efficiency of hormones from new leaves to flower buds in genotype 80 than genotype 195.

### Transcriptome profiling of leaves and flower buds

**Fig. 3:**
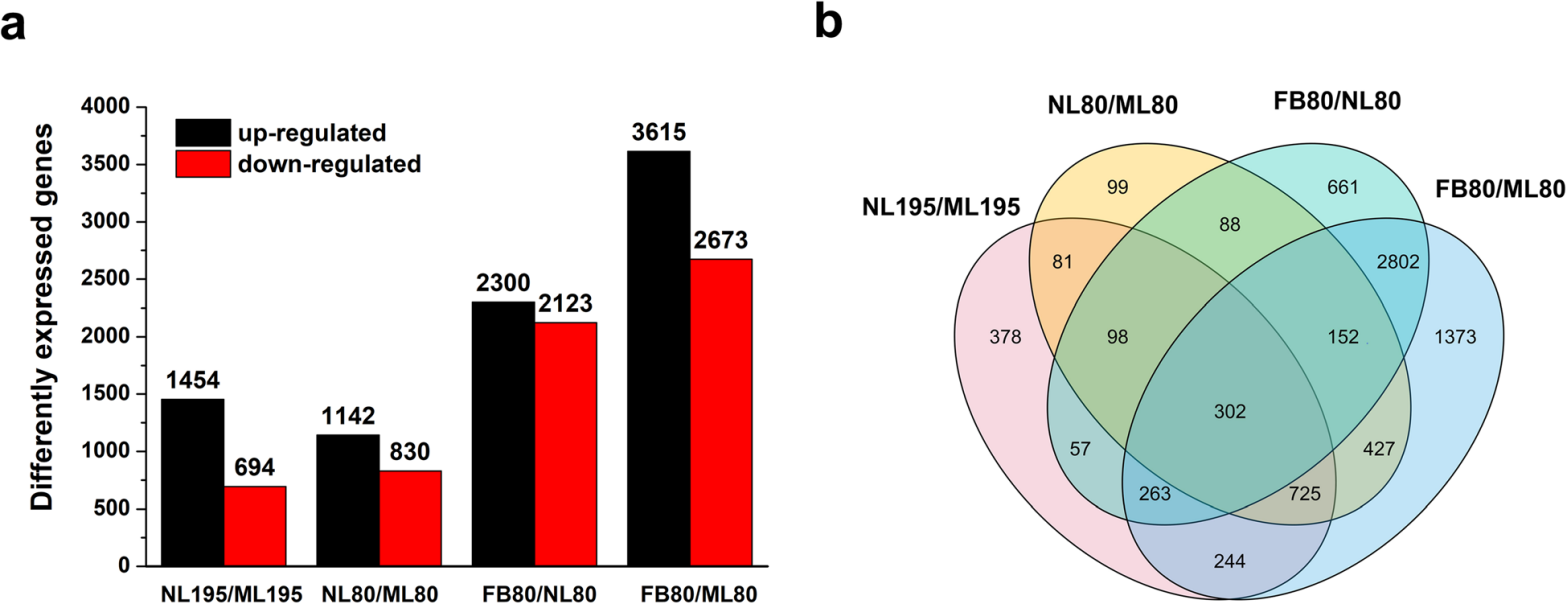
The bar chart (**a**) and Venn diagram (**b**) of differentially expressed genes (DEGs) in genotypes 195 and 80

To further investigate the molecular basis of the early-flowering phenotype in genotype 80, we performed transcriptome profiling in the ML, NL, and FB of both genotypes (Table 1). A total of 101.13 Gb of clean reads were obtained, the Q30 percentage (the percentage of bases with a quality score of 30 or higher) and GC (guanine and cytosine) percentage were 93.69 and 42.28%, respectively. A total of 53,897 unigenes were yielded from the assembly (N50 of 1770 bp, mean length of 1106 bp) and were annotated using seven functional databases, including the NCBI non-redundant protein database (NR), Nucleotide sequence database (NT), Clusters of Orthologous Groups of proteins (COG), Gene Ontology (GO) and Kyoto Encyclopedia of Genes and Genomes (KEGG), Swissprot and Interpro (E-value < 10^− 5^). Then, 31,962 coding sequence transcripts (N50 of 1866 bp, mean length of 1336 bp) were used for further functional analysis (Table 1). We identified 2148 (1454 upregulated/694 downregulated) and 1972 (1142 upregulated/830 downregulated) differentially expressed genes (DEGs) between NL and ML in 195 and genotype 80 respectively, among which 942 and 766 DEGs were exclusively expressed in genotype 195 and 80, respectively (Fig. 3a and b; Additional file 1: Table S1). In addition, 4423 (2300 upregulated/2123 downregulated) DEGs were identified between FB and NL in genotype 80, and 6288 (3615 upregulated/2673 downregulated) DEGs were identified between FB and ML in genotype 80, among which 2802 common DEGs were found to respond to early flowering in genotype 80 (Fig. 3a and b; Additional file 1: Table S1).

**Table 1:** Summary of the transcriptome analysis of alfalfa leaves and flower buds

Total clean reads (Gb)	101.13
Q30 bases (%)	93.69
GC content (%)	42.28
Total number of unigenes	53,897
N50 of unigenes (bp)	1770
Mean length of unigenes (bp)	1106
Number of transcripts (coding sequence, CDS)	31,962
N50 of transcripts (bp)	1866
Mean length of transcripts (bp)	1336

### Response of hormone-related genes to early flowering

**Fig. 4:**
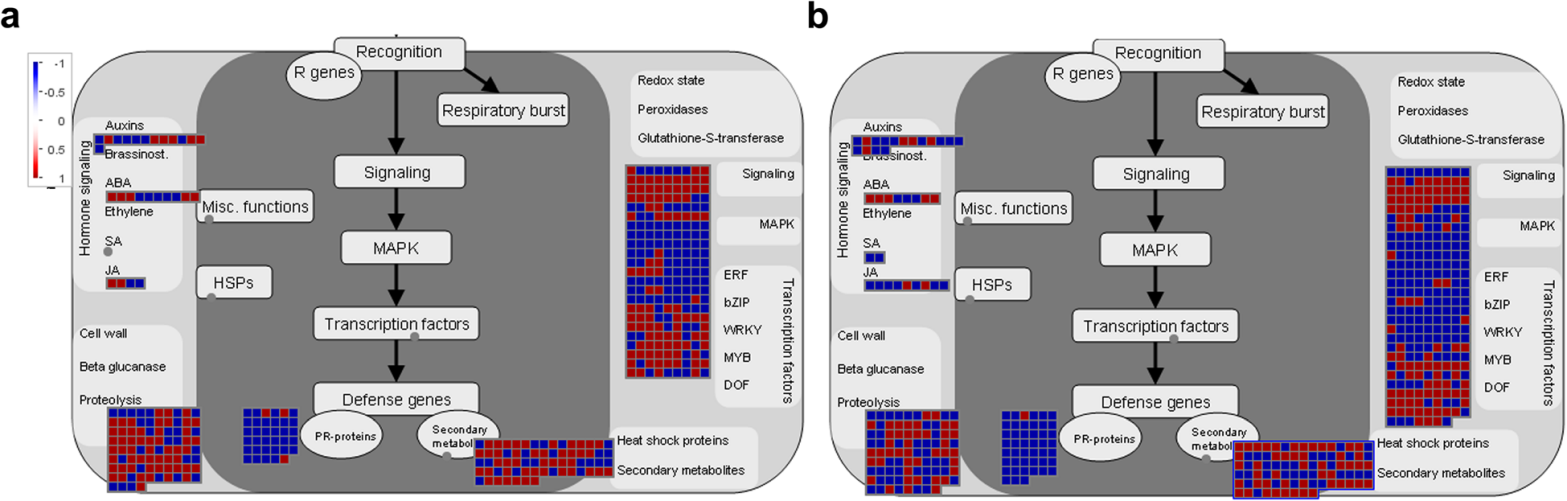
MapMan display of the functional categories of DEGs between NL and ML in genotypes 195 (**a**) and 80 (**b**) upon the flowering of genotype 80. Squares represent DEGs; red and blue indicate up- and downregulated genes, respectively

**Fig. 5:**
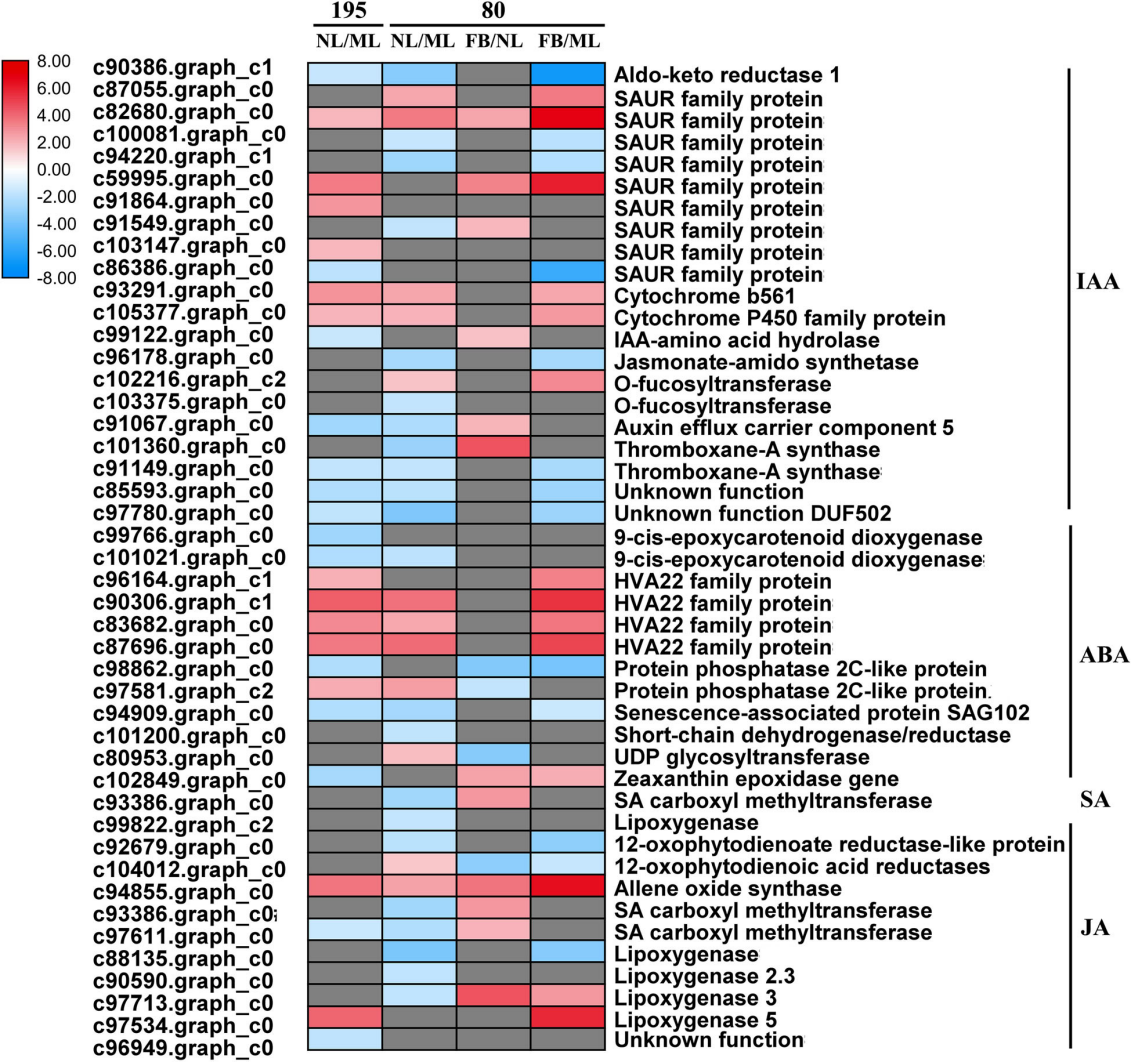
Transcript profiles of hormone-related genes in genotypes 195 and 80. The color scale indicates log2-transformed fold changes in gene expression levels in NL compared with ML, and in FB compared with NL or ML in genotypes 195 and 80. Red, blue, and gray denote upregulation, downregulation, and no change in expression, respectively

The significant change in hormone levels prompted us to further examine the transcript levels of hormone-related genes. The analysis detected 27 and 34 DEGs (between NL and ML) involved in the IAA, ABA, SA and JA metabolism and signaling pathways in genotype 195 (NL195/ML195) and genotype 80 (NL80/ML80), respectively (Figs. 4a, b and 5; Additional file 2: Table S2). Among these DEGs, 11 of the 16 IAA metabolism and signaling pathway genes in NL were significantly downregulated compared with ML in genotype 80 (Fig. 5; Additional file 2: Table S2). Seven of the 13 IAA-related genes were downregulated in NL compared with ML in genotype 195, such as those encoding SAUR family proteins, o-fucosyltransferase, auxin efflux carrier component 5, and thromboxane-A synthase (Fig. 5; Additional file 2: Table S2). Furthermore, ten DEGs in genotype 195 (five downregulated/five upregulated) and eight DEGs in genotype 80 (three downregulated/five upregulated) were enriched in NL compared with ML (Fig. 5; Additional file 2: Table S2). These were primarily involved in ABA metabolism and signaling pathways, including the ABA-responsive protein, 9-cis-epoxycarotenoid dioxygenase, and HVA22-like protein encoding-genes (Fig. 5; Additional file 2: Table S2). Two SA-related genes that encode SA carboxyl methyltransferase (SAMT) and lipoxygenase were significantly downregulated in NL compared with ML in genotype 80 but were not enriched in genotype 195 (Fig. 5; Additional file 2: Table S2). Seven of the nine JA biosynthetic genes were significantly downregulated in NL compared with ML in genotype 80; while two of the four JA-related genes were downregulated in NL compared with ML in genotype 195, including those encoding 12-oxophytodienoate reductase-like protein, allene oxide synthase, and lipoxygenase (Fig. 5; Additional file 2: Table S2). These results further indicate that the downregulation of these hormone-related genes may have a negative impact on early flowering in genotype 80. In addition, 16 and 27 DEGs related to hormone metabolism and signaling pathways were identified between FB and NL and between FB and ML, respectively, in genotype 80; these include genes involved in ABA signal transduction and genes encoding auxin-responsive proteins, SAMT, allene oxide synthase, and lipoxygenase 3 (Fig. 5; Additional file 2: Table S2).

### Response of genes- related to pathogenesis and metabolism to early flowering

**Fig. 6:**
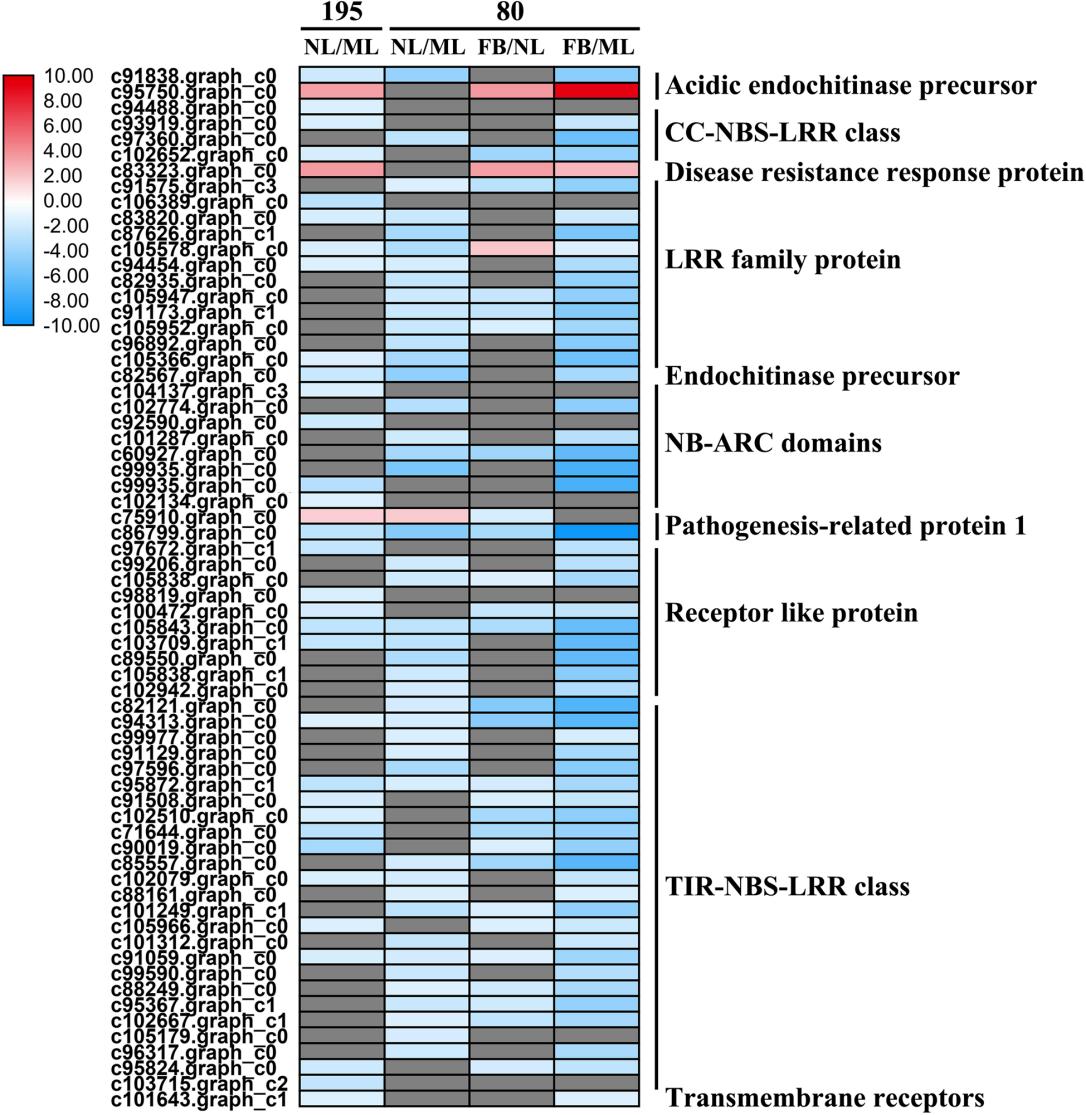
Transcript profiles of pathogenesis-related genes in genotypes 80 and 195. The color scale indicates log2-transformed fold changes in expression levels of NL compared with ML, and FB compared with NL or ML in genotypes 195 and 80. Red, blue, and gray denote upregulation, downregulation, and no change in expression, respectively

Thirty-five DEGs (three upregulated/32 downregulated) in genotype 195 and 45 DEGs (one upregulated/44 downregulated) in genotype 80 were significantly enriched in NL compared with ML. These mainly include genes involved in the Toll-interleukin-like receptor (TIR)-nucleotide-binding site (NBS)-LRR and coiled-coil (CC)-NBS-LRR classes and those encoding LRR family proteins, NB-ARC (nucleotide-binding adaptor shared by APAF-1, R proteins, and CED-4) domain-containing proteins, and receptor-like proteins (Figs. 4a, b and 6; Additional file 2: Table S2). Meanwhile, 29 (three upregulated and 26 downregulated) DEGs between FB and NL and 57 (two upregulated and 55 downregulated) DEGs between FB and ML were identified in genotype 80 (Additional file 2: Table S2). We found that 66 metabolic DEGs between NL and ML (43 upregulated and 23 downregulated) were significantly enriched in genotype 195; whereas 82 such DEGs (47 upregulated and 35 downregulated) were enriched in genotype 80. These mainly include genes encoding 2-oxoglutarate and Fe (II)-dependent oxygenase superfamily proteins (2OG oxygenases), chalcone synthase, laccases, and UDP-Glycosyltransferase superfamily proteins (UGTs) (Figs. 4a, b and 6; Additional file 2: Table S2). Fifty (40 upregulated and 10 downregulated) DEGs between FB and NL and 78 (50 upregulated and 28 downregulated) DEGs between FB and ML were identified in genotype 80 (Additional file 2: Table S2). Altogether, these findings indicate that compared with genotype 195, more PR genes were downregulated, and more metabolism-related genes were upregulated in genotype 80 upon flowering.

### Responses of receptor-like kinase family genes to early flowering

**Fig. 7:**
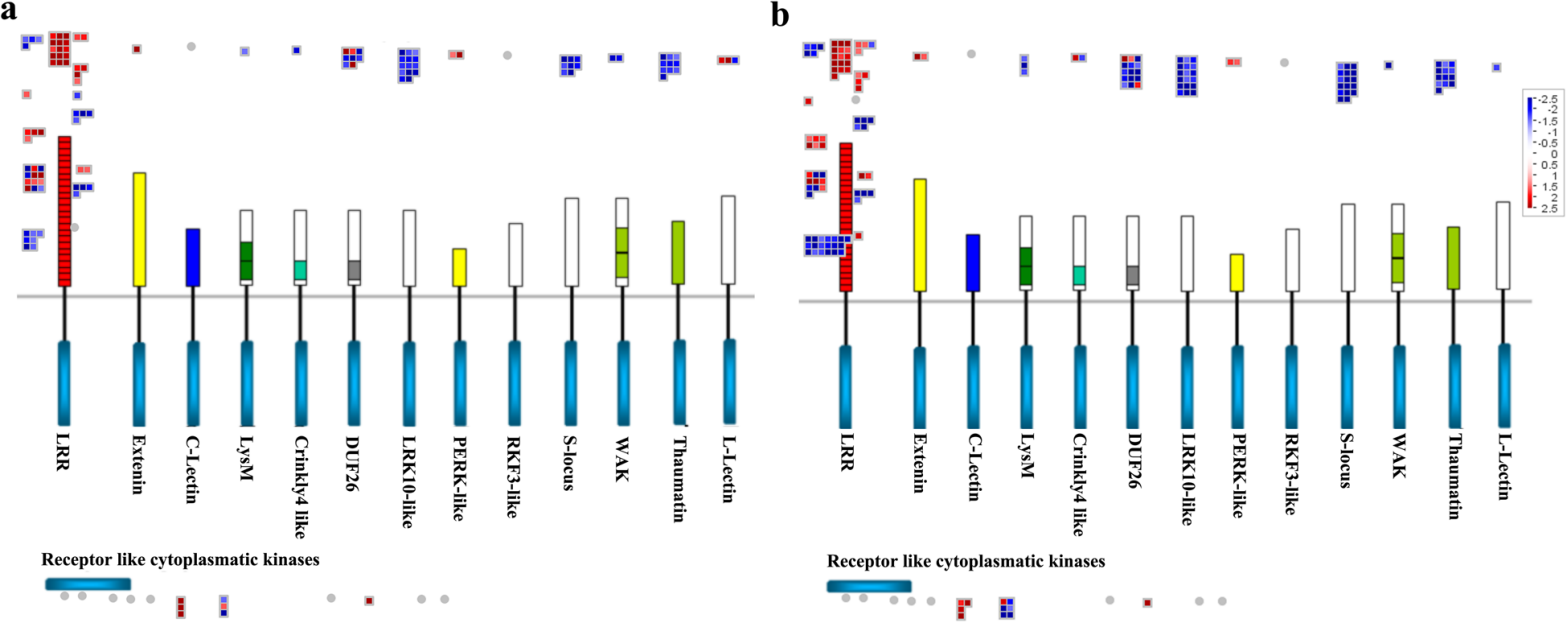
MapMan display of the coordinated changes in the expression levels of genes involved in the receptor kinase signaling pathway in genotypes 195 and 80 upon the flowering of genotype 80. Shown are DEGs between NL and ML in genotypes 195 (**a**) and 80 (**b**). Squares represent DEGs; red and blue indicate up- and downregulated genes, respectively

Furthermore, we observed differences in the expression of receptor-like kinase family genes in response to early flowering. For example, 117 DEGs between NL and ML (48 up-regulated and 69 down-regulated) were identified in genotype 195, and 162 such DEGs (52 up-regulated and 110 down-regulated) were identified in genotype 80. These mainly include genes encoding cytoplasmatic kinases, LRR-kinases, the DUF 26 (domain of unknown function 26) receptor-like kinase, the wheat LRK10 (leucine-rich repeat receptor-like kinase10)-like receptor kinase, and S-locus glycoprotein-like kinases (Figs. 4a, b and 7; Additional file 2: Table S2). Meanwhile, 83 DEGs between FB and NL (24 up-regulated and 59 down-regulated) and 177 DEGs between FB and ML (57 up-regulated and 120 down-regulated) were identified in genotype 80 (Additional file 2: Table S2). Taken together, these results reveal that more receptor-like kinase genes were downregulated in response to early flowering in genotype 80 than in genotype 195.

### Response of proteasome-related genes to early flowering

**Fig. 8:**
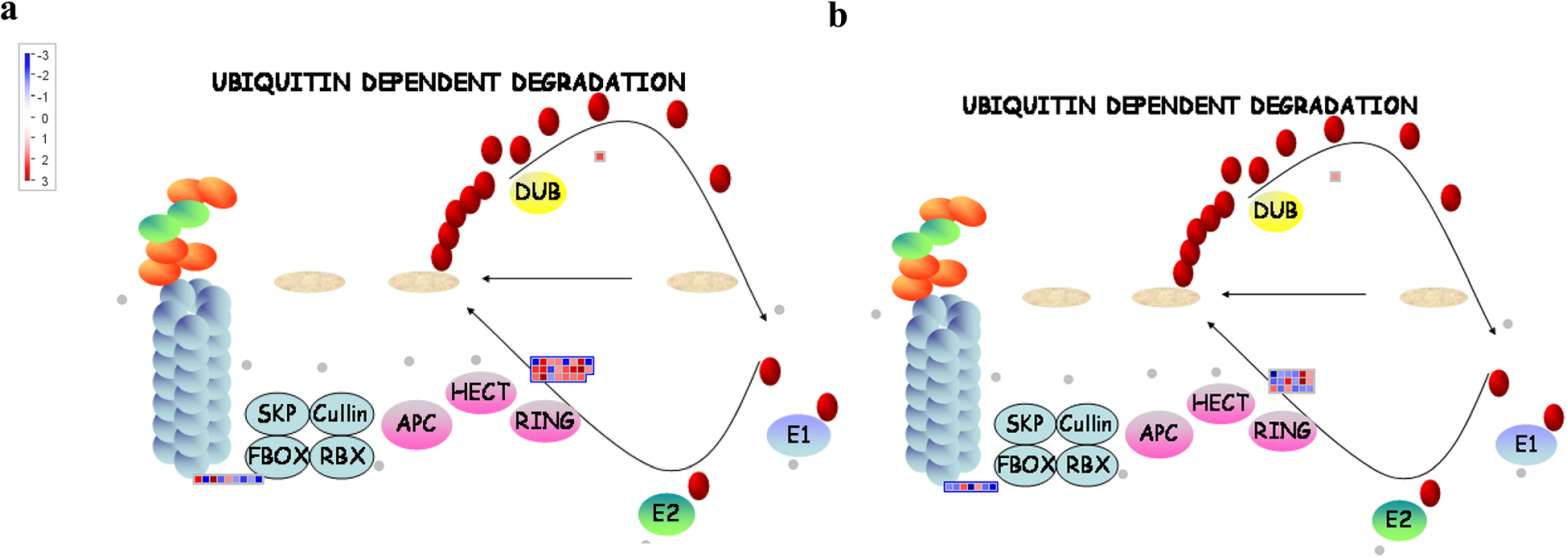
MapMan display of coordinated changes in the expression levels of genes involved in the ubiquitin-dependent degradation pathway in genotypes 195 and 80 upon the flowering of genotype 80. Shown are DEGs between NL and ML in genotypes 195 (**a**) and 80 (**b**). Squares represent DEGs; red and blue indicate up- and downregulated genes, respectively

**Fig. 9:**
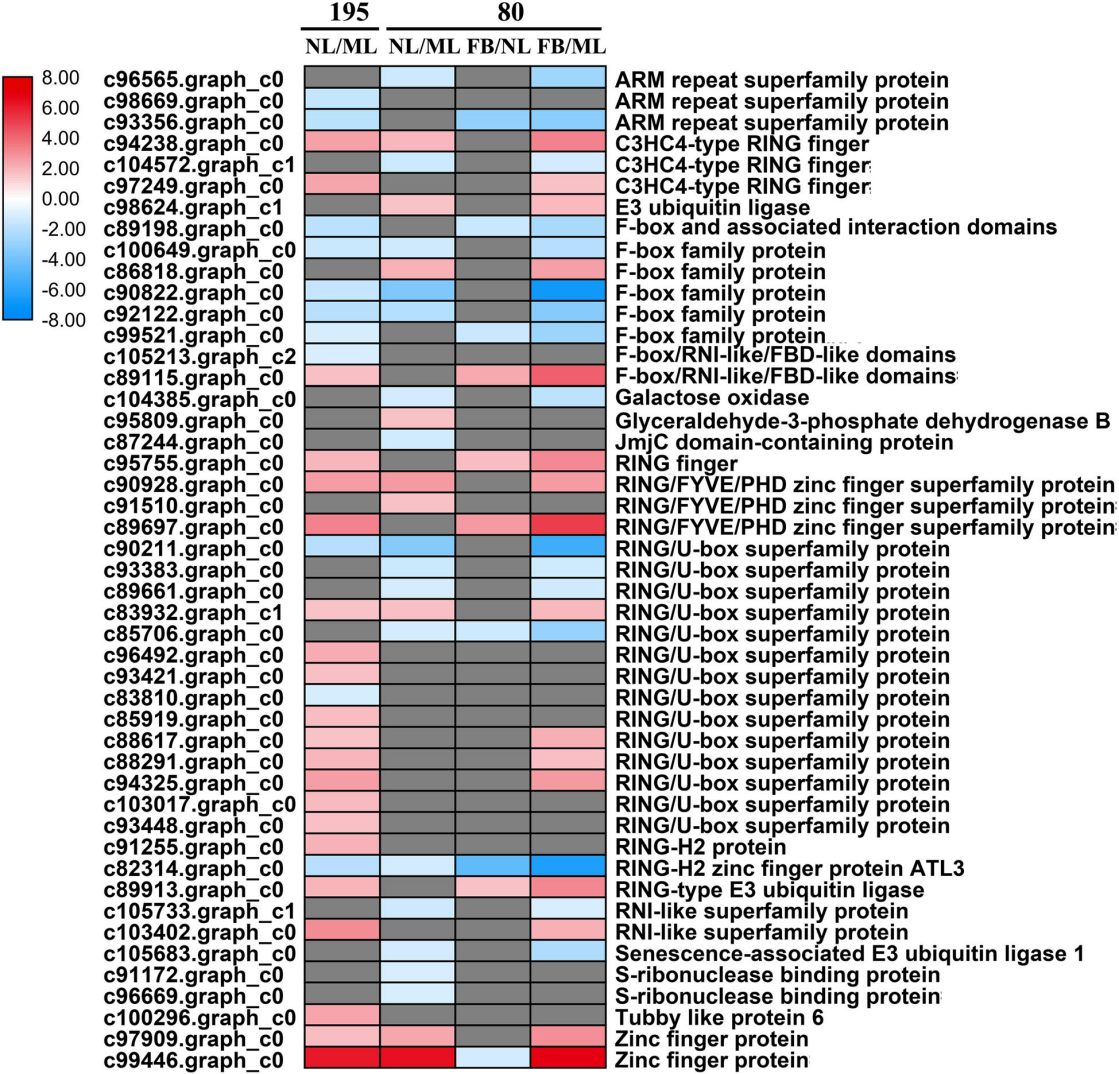
The transcript profiles of genes involved in the ubiquitin-dependent degradation pathway in genotypes 80 and 195. The color scale indicates log2-transformed fold changes in gene expression levels in NL compared with ML and in FB compared with NL or ML in genotypes 195 and 80. Red, blue, and gray denote upregulation, downregulation, and no change in expression, respectively

Thirty-two proteasome-related DEGs between NL and ML (21 up-regulated and 11 down-regulated) were identified in genotype 195, and 25 such DEGs (nine upregulated and 16 downregulated) DEGs were identified genotype 80. These genes mainly encode F-box family proteins, really interesting new gene (RING) /U-box superfamily proteins, and S-ribonuclease binding proteins (Fig. 4a, b and 8; Additional file 2: Table S2). Meanwhile, ten DEGs between FB and NL (four up-regulated and six down-regulated) and 32 DEGs between FB and ML (16 upregulated and 16 downregulated) were identified in genotype 80 (Figs. 8 and 9; Additional file 2: Table S2). Together, these results reveal that more proteasome related genes were downregulated in response to early flowering in genotype 80.

### qRT-PCR validation of DEGs identified by RNA-seq

**Fig. 10:**
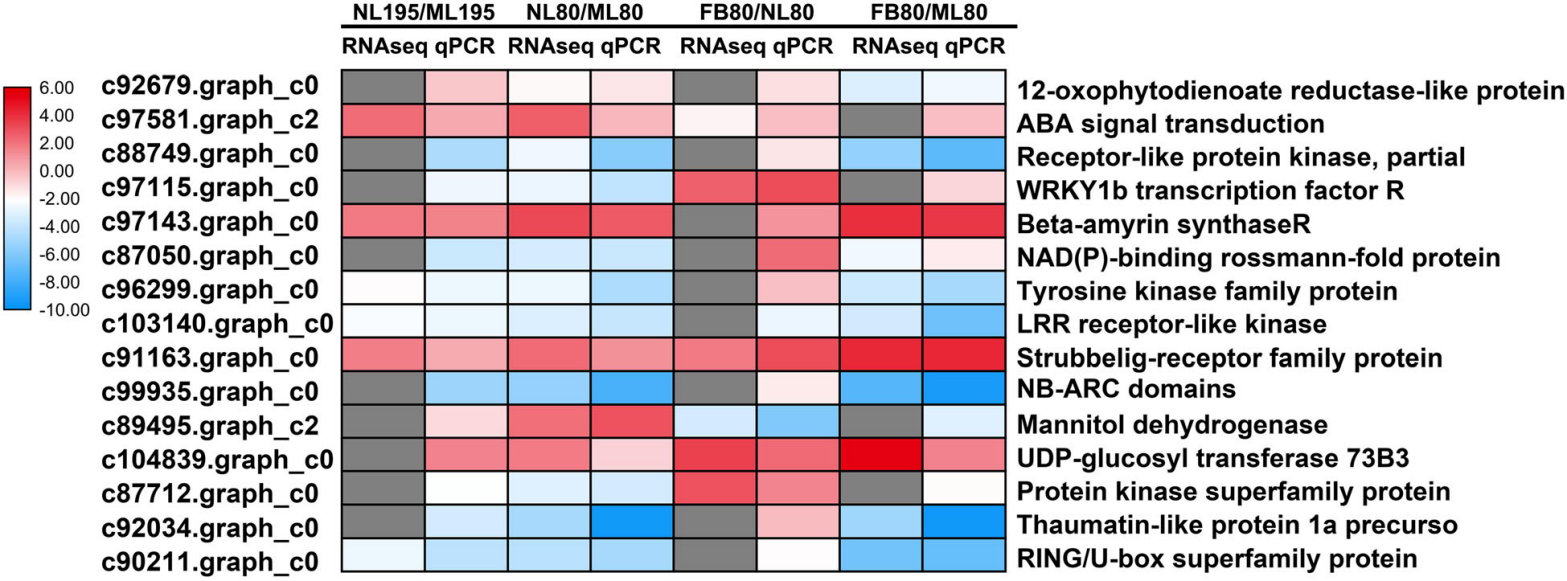
Comparison between the results of the qRT-PCR and RNA-seq analyses of selected DEGs. The color scale indicates log2-transformed fold changes in gene expression levels. Red, blue, and gray denote upregulation, downregulation, and no change in expression, respectively

We randomly selected 15 DEGs between NL and ML identified by transcriptome profiling from genotypes 195 for qRT-PCT validation. Six of the 15 DEGs showed good agreement between the qRT-PCR analysis and the RNAseq data. These include genes involved in ABA signal transduction, and those encoding beta-amyrin synthase R, a tyrosine kinase family protein, an LRR receptor-like kinase, and a RING/U-box superfamily protein. For genotype 80, the expression of all DEGs between NL and ML exhibited a high correlation between the RNAseq data and qRT-PCR analysis; similarly, six DEGs between FB and NL and 11 DEGs also showed good agreement between the RNA-seq data and qRT-PCR analysis (Fig. 10; Additional file 3: Table S3).

## Discussion

### Early flowering of alfalfa is related to hormone metabolism

Hormones play distinctive roles in controlling plant growth and development, especially in flowering processes [24, 25]. For instance, disruption of auxin biosynthesis, signaling, or polar auxin transport can extensively inhibit the initiation of inflorescence [15, 26] whereas exogenous ABA or JA applications can significantly delay floral transition [13, 14]. Exogenous SA is known to induce flowering by suppressing *FLC* expression and increasing *FT*, *CO*, and *SOC1* expression in tobacco (*Nicotiana tabacum* L.) [27], *Lemna paucicostata* [28] and Arabidopsis [29]. In the present study, the ratio of hormone contents (IAA, ABA, SA, and JA) in new leaves compared with mature leaves was lower in the early-flowering genotype 80 compared with the late-flowering genotype 195. Consistent with this observation, 11 auxin-related genes were downregulated in NL compared with ML in phenotype 80, these include those encoding SAUR family proteins, o-fucosyltransferase, auxin efflux carrier component 5, and thromboxane-A synthase. Seven JA-related genes were downregulated in NL compared with ML in genotype 80, including those encoding the lipoxygenase, the 12-oxophytodienoate reductase-like protein, and SAMT. Meanwhile, we observed the upregulation of genes involved in JA biosynthesis in the FB of genotype 80, such as genes encoding SAMT and lipoxygenase3/5. In addition, we detected higher ABA and SA contents as well as higher transcript levels of the HVA22 family protein and SAMT encoding genes in the FB of genotype 80 compared with genotype 195. ABA is known to induce AtHAV22 expression mainly in flower buds and inflorescence tissues, whereas SAMT catalyzes the production of methyl-salicylate from SA, which confers pathogen resistance in Arabidopsis and white tea (*Camellia sinensis* L.) [30–32]. Therefore, the early-flowering phenotype of genotype 80 might be negatively regulated by JA biosynthetic genes, and the interactive networks of these hormones may also contribute to early flowering in alfalfa.

### The early flowering of alfalfa is associated with pathogenesis-related and signaling receptor kinase genes

Plants often respond to biotic and abiotic stress by diverting more resources away from growth and development to promote early flowering [9]. For instance, Arabidopsis plants infected by fungal and bacterial pathogens showed an accelerated flowering time and increased transcript levels of floral integrator genes *FT* and *GIGANTEA*. Further investigation indicated that 19 genes encoding receptor-like proteins were upregulated in the Arabidopsis-*Fusarium oxysporum*-resistant mutants [9, 33]. In the present study, 97% of PR genes were downregulated in the new leaves of genotype 80, and the genes encoding 11 LRR family proteins, seven receptor-like proteins, and 18 TIR-NBS-LRR class proteins were significantly downregulated. In addition, 67.9% of the genes involved in the kinase signaling pathway were downregulated in genotype 80 compared with genotype 195, including 38 of the 74 LRR proteins, 12 of the 15 DUF26 kinases, 30 wheat LRK10-like kinases, and 16 S-locus glycoprotein-like kinases. Previous studies have demonstrated that the overexpression of *TaBRI1* (*BRASSINOSTEROID*-*INSENSITIVE1*) and *ERECTA* lead to early flowering and increased slique size in Arabidopsis and tomato, respectively, and overexpression of the tomato LRR receptor-like kinase enhances drought tolerance [34, 35]. In addition, overexpression of *AtLRK10L1.2*, the homolog of wheat LRK10-like kinase, confers positive response toward ABA signaling and drought tolerance, features associated with early flowering in Arabidopsis [16]. In line with these published results, we found that genotype 80 suffered from pathogen attacks and abiotic stress while exhibiting early flowering during our field experiments. Consistently, pathogenesis-related and signaling receptor kinase genes were significantly downregulated in genotype 80 compared with the later-flowering genotype 195.

### The early flowering of alfalfa is related to secondary metabolism and protein degradation

2-oxoglutarate/Fe (II)-dependent dioxygenases play versatile roles in secondary metabolite synthesis and catalyze epimerization and demethylation during floral development [36, 37]. For example, *PKDM78* and *JMJ14*, which 2OG oxygenases, suppress the expression of floral integrator genes *FT*, *AP1*, *SOC1*, and *LFY* by mediating H3K4 demethylation of chromatin, thereby inhibiting floral transition in Arabidopsis [38, 39]. In this study, eight of ten 2OG oxygenases were significantly downregulated in the new leaves of genotype 80, indicating that 2OG oxygenases might negatively affect early-flowering. Furthermore, a previous study reported that glycosyltransferases catalyze the glycosylation of plant secondary compounds to maintain cell homeostasis and regulate plant growth and development [40]. Ectopic overexpression of glycosyltransferase genes *UGT87A2* and *PtGT1* were found to inhibit *FLC* expression and increase floral integrator gene expression, thus resulting in an early-flowering phenotype in Arabidopsis and tobacco, respectively [40, 41]. Here, seven UDP-glycosyltransferases were significantly upregulated in the new leaves of genotype 80 compared with those of genotype 195; these genes were also significantly upregulated in the flower buds compared with mature or new leaves in genotype 80. Together, these results indicate the *UGT* genes might play critical roles in regulating early flowering in genotype 80. The widely existed ubiquitin/proteasome system affects plant development by participating in signaling transduction cascades, pathogen defense, and biotic and abiotic stress response [19]. For instance, HOS1 (high expression of osmotically responsive genes1), a RING finger-containing E3 ubiquitin ligase, negatively regulates CONSTANS abundance and delays flowering time in Arabidopsis [42]. By contrast, the FKF1 F-box protein physically interacts with CDF1 (CYCLING DOF FACTOR 1) to induce its degradation, thereby increasing the expression of CO, which promotes early-flowering in Arabidopsis [43]. In addition, the F-box protein *UFO* (*UNUSUAL FLORAL ORGANS*) associates with the AP3 promoter to enhance *LFY* transcription [44]. Consistent with these published data, we found that nine of the 11 RING/U-box superfamily proteins were significantly upregulated in new leaves in genotype 195 compared with genotype 80, indicating that these ubiquitin/proteasome-related genes may be negative regulators of flowering time. Six of seven F-box family proteins were significantly downregulated in the new leaves of genotype 195 compared with genotype 195, suggesting that these F-box proteins may promote early flowering in alfalfa. These findings suggest that secondary metabolism genes and protein degradation genes may be essential for flowering time regulation in alfalfa.

## Conclusions

**Fig. 11:**
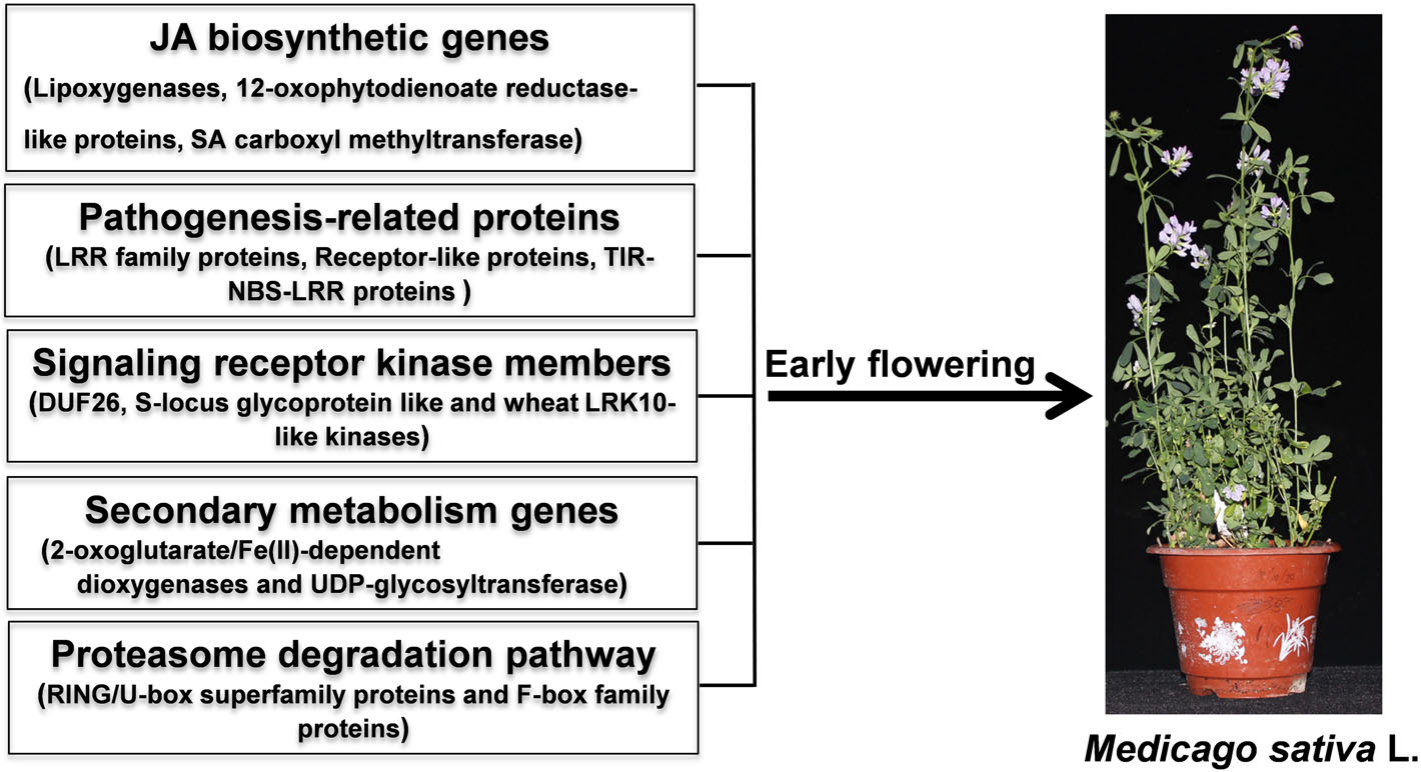
Diagram of a proposed regulatory network for early flowering in alfalfa

Based on the phenotypical, physiological and transcriptomic analyses, reduced JA content in new leaves and the downregulation of JA biosynthetic genes, including those encoding lipoxygenases, 12-oxophytodienoate reductase-like proteins, and SAMT may play essential roles in controlling the early-flowering phenotypes in alfalfa. Further analyses reveal that *PR* genes (including those encoding LRR family proteins, receptor-like proteins, and TIR-NBS-LRR class proteins), genes encoding signaling receptor kinase family members (including DUF26 and wheat LRK10-like kinases), secondary metabolism genes that encode 2OG oxygenases and UGTs, and proteasome degradation pathway genes (such as those encoding RING/U-box superfamily proteins and F-box family proteins) also contribute to the establishment of early-flowering in alfalfa (Fig. 11). In summary, our results provide insights into the regulatory mechanisms underlying early flowering in alfalfa and offer new target genes for future functional characterization and the genetic improvement of alfalfa.

## Methods

### Plant materials and growth conditions

Alfalfa genotype 80 (Accession No. PI183404, collected in India) and genotype 195 (Accession No. PI584512, collected in Canada) were provided by the USDA National Plant Germplasm System as previously described [45]. Seedlings were transplanted to the Shangzhuang experimental station of China Agricultural University, Beijing (116.32^o^ E, 40.14^o^ N) in 2018. Flowering time investigations for two consecutive years showed that genotype 80 began to flower 20 days earlier than genotype 195 in 2018 and 2019. We propagated their clonal lines from cuttings and grew them in a controlled climate growth chamber at 25°/23 °C (day/night), with a 16-h photoperiod, 650 mmol m^−2^s^−1^light intensity, and 60% relative humidity. Three replicates were arranged in a randomized complete block design, each replicate contained three plants (from the same clone of the unanimous genotype) grown in individual pots.

### Total RNA extraction, RNA-Seq library construction and sequencing

When genotype 80 first flowered, mature leaves, new leaves (including apical meristem), and flower buds were collected from three individual plants in each replicate for both genotypes 80 and 195. The harvested tissues were immediately frozen in liquid nitrogen and stored at -80 °C for RNA extraction. Total RNA was extracted using the Trizol reagent (Invitrogen, USA) according to the manufacturer’s instructions. After DNase treatment, the quality and concentration of the RNA were determined using the Agilent Bioanalyzer 2100 system (Agilent Technologies, USA), samples with a 260:280 ratio of ≥2.0 and RNA integrity number (RIN) of ≥8 were subjected to transcriptome sequencing. RNA libraries were generated using the NEBNext®Ultra™ RNA Library Prep Kit for Illumina® (NEB, USA) following the manufacturer’s recommendations. The libraries were sequenced on an Illumina Hiseq 2000 platform (Illumina, USA) to generate paired-end reads. For quality control, raw reads of the FASTQ format were processed through in-house Perl scripts, after which reads containing adapter sequences were removed and 100.13 Gb of clean reads for fifteen alfalfa samples were obtained. Next, the Q30, GC-content, and sequence duplication level of the clean reads were calculated. All downstream analyses were performed on the clean reads with high quality. The de novo assembly of non-redundant unigenes was performed by using the trinity program (v20131110) [46].

### Functional annotation of unigenes and analysis of differentially expressed genes

Functional annotation of the unigenes was performed by BLAST search against public databases, including NR, NT, protein family (Pfam), COG, Swiss-Prot, GO, and KEGG considering E-value threshold ≤10^− 5^. The identification of DEGs between different tissues was performed using the DESeq R package (v1.10.1) [47], and genes with a |log2 fold change| > 1.5 and an adjusted *P*-value < 0.05 were considered as differentially expressed. For the classification and functional analysis of identified proteins, their homologs were identified using the Phytozome *Medicago truncatula* genome database (https://phytozome.jgi.doe.gov/pz/portal.html#!info?alias=Org_Mtruncatula, Mt4.0v1, E-value < 10^− 5^), and the MapMan software (https://mapman.gabipd.org/download, V3.5.1R2) was used for flowering-time associated regulatory network analysis. The Venn graph and heat maps were drawn using TBtools (https://github.com/CJ-Chen/TBtools/releases).

### Endogenous ABA, IAA, JA, and SA analyses in alfalfa

Endogenous hormone contents were determined in the leaves (the same leaves as those used for the RNA-seq analysis) of genotypes 80 and 195. ABA, IAA, JA, and SA were extracted and purified using previously reported methods [45, 48]. Briefly, ~ 50 mg of frozen tissue was ground in liquid nitrogen and mixed with 500 μL of indolepropionic acid extraction buffer (2:1 dilution) and 50 μL of internal standards (^2^H_6_ ABA, ^2^H_2_ IAA, ^2^H_5_ JA, and ^2^H_4_ SA). The mixture was centrifuged at 15000 g for 5 min, the supernatant was mixed with 1 mL chloroform, and dissolved in 100 μL methanol and used for liquid chromatography coupled with tandem mass spectrometry (LC-MS) according to Cao et al. [48] . The content of each hormone was calculated according to the standard curve of the internal standards and normalized to fresh weight (FW) associated with three biological replicates.

### qRT-PCR validation of sixteen DEGs

Total RNA was isolated from the same samples used for the RNA-seq analysis using an RNAprep Pure Plant Kit (Tiangen, Beijing, China) following the manufacturer’s instructions. First-stand cDNA was synthesized using the Takara MLV-Reverse transcriptase (Takara Bio, Inc., Otsu, Japan) used for qRT-PCR analysis on the Bio-Rad CFX96 real-time PCR detection system with four biological replicates and using the SYBR Premix Ex Taq (Takara Bio, Inc., Otsu, Japan). Sixteen unigenes were randomly selected for the qRT-PCR analysis using gene-specific qRT-PCR primers (Additional file 3: Table S3). The 2^-ΔΔCT^ method [49] was used to calculate the relative expression level of each gene to the endogenous control *Actin*.

### Statistical analysis

All data were subjected to the analysis of variance (ANOVA) in SAS 9.0 (SAS Institute, Cary, NC) using the general linear model. Fisher’s least significant difference test (LSD, *P* < 0.05 was used to determine significant differences among treatments according to Ma et al. [50].

## Supplementary Information

**Additional file 1: Table S1.** Annotation of DEGs identified in genotypes 195 and 80.

**Additional file 2: Table S2.** Mapman display of the functional categories of DEGs in genotypes 195 and 80.

**Additional file 3: Table S3.** qRT-PCR validation of selected DEGs identified by RNA-seq in genotypes 195 and 80.

## Data Availability

The RNA-seq data have been deposited into NCBI under accession number PRJNA602419 (https://dataview.ncbi.nlm.nih.gov/object/PRJNA602419).

## Abbreviations

FT: FLOWERING LOCUS T
FLC: FLOWERING LOCUS C
CO: CONSTANS
SOC1: SUPPRESSOR OF OVEREXPRESSION OF CONSTANS1
SPL: SQUAMOSA PROMOTER BINDING-LIKE
JA: Jasmonate-activated
ABA: Abscisic acid
ABI5: ABSCISIC ACID-INSENSITIVE MUTANT 5
RLK: Receptor-like kinase
LRR: Leucine-rich repeat
PR: Pathogenesis-related
IAA: Auxin
SA: Salicylic acid
SAMT: SA carboxyl methyltransferase
TIR-NBS-LRR: Toll-interleukin-like receptor -nucleotide-binding site-LRR
CC-NBS-LRR: Coiled-coil-NBS-LRR
NB-ARC: Nucleotide-binding adaptor shared by APAF-1, R proteins, and CED-4
2OG oxygenases: 2-oxoglutarate and Fe (II)-dependent oxygenase superfamily proteins
BRI1: BRASSINOSTEROID-INSENSITIVE1
CDF1: CYCLING DOF FACTOR 1
UFO: UNUSUAL FLORAL ORGANS
UGTs: UDP-Glycosyltransferase superfamily proteins
DUF 26: Domain of unknown function 26
